# Low density lipoprotein cholesterol is inversely correlated with abdominal visceral fat area: a magnetic resonance imaging study

**DOI:** 10.1186/1476-511X-10-12

**Published:** 2011-01-19

**Authors:** Michel R Hoenig, Gary Cowin, Raymond Buckley, Christine McHenery, Allan Coulthard

**Affiliations:** 1 Royal Brisbane and Women's Hospital, University of Queensland Centre for Clinical Research, Brisbane, Queensland, Australia; 2 University of Queensland, Brisbane, Queensland, Australia; 3 Department of Medical Imaging, Royal Brisbane and Women's Hospital and University of Queensland, Brisbane, Queensland, Australia

## Abstract

**Background:**

Visceral Fat Area (VFA) is an independent predictor of coronary disease. While low density lipoprotein cholesterol (LDL-C) is used to determine risk and guide therapy, its accuracy fails in obese patients who may have low LDL-C despite high VFA.

**Objective:**

We sought to describe the relationship between VFA, LDL-C and to describe shifting cholesterol metabolism with increasing VFA.

**Methods:**

42 High-risk vascular patients not on lipid-lowering therapy provided a fasting lipid profile and underwent magnetic resonance imaging (MRI) to quantify VFA and subcutaneous fat area (SFA) at the L4-L5 disc. Comparisons: 1. Correlation between VFA, SFA, LDL-C and the standard lipid panel 2. Correlation between VFA, SFA and markers of cholesterol synthesis (desmosterol, lathosterol) and cholesterol absorption (cholestanol, sitosterol).

**Results:**

VFA was inversely correlated with LDL-C (r = -0.348) indicating potential discordance between cardiovascular risk and LDL-C. However, VFA was appropriately correlated with other markers of increased risk: r = -0.361 with HDL-C, r = 0.503 with VLDL-C, r = 0.499 with TG (all p < 0.05). VFA did not correlate significantly with non-HDL-C. VFA correlated positively with cholesterol synthesis markers (desmosterol, lathosterol) and negatively with an absorption marker (cholestanol).

**Conclusions:**

LDL-C is inversely correlated with VFA and this may explain the loss of the relationship between LDL-C and cardiovascular events in the obese. While Non-HDL-C did not correlate positively with VFA, the absence of a negative correlation suggests that it may be a more appropriate lipid target in an increasingly obese world.

## Introduction

Visceral fat area (VFA), measured at the umbilicus, has been independently linked to the development of coronary artery disease (CAD) while subcutaneous fat area (SFA) has not been shown to carry prognostic significance [1]. The accumulation of visceral adipose tissue is associated with the adverse metabolic consequences of obesity and VFA is a better predictor of cardiovascular risk factors than body mass index (BMI), even in non-obese individuals [1-6]. However, in determining patient risk and tailoring therapy, clinicians rarely quantify fat areas using imaging modalities and typically rely on the standard lipid panel. The current target of therapy is low density lipoprotein cholesterol (LDL-C) which shows a log-linear relationship with cardiovascular events in both primary and secondary prevention [7]. However, several reports have highlighted the loss of the relationship between LDL-C and subsequent cardiovascular events, as reviewed previously [6]. In a cohort from the Lipid Research Clinics prevalence study, men with LDL-C <100 mg/dl, had an increased cardiovascular mortality when compared to men with LDL-C in the 100-130 mg/dl range and hence the log-linear relationship between LDL-C and coronary events appears invalid, especially in patients with high triglycerides (TG) >200 mg/dl [8]. Similarly, an analysis of diabetic patients (average TG 254 mg/dl) also suggests a dissociation between CAD death and LDL-C [9]. In a large cohort of patients with CAD, diabetes was identified as a predictor of lower LDL-C levels; despite diabetics being at higher risk for cardiovascular events [10]. Indeed, the epidemiological studies on the basis of which the Adult Treatment Panel III (ATPIII) formulated guidelines were from an era when diabetes, obesity and the metabolic syndrome were less prevalent [6].

LDL-C has been criticized as a target of therapy that does not take into account very low-density lipoprotein cholesterol (VLDL-C) which predicted coronary events independently of LDL-C in the Framingham cohort [11]. The current ATPIII guidelines only recommend the consideration of the VLDL-C fraction in patients with hypertriglyceridemia. Non-High density lipoprotein cholesterol (non-HDL-C), calculated as total cholesterol minus high density lipoprotein cholesterol (TC - HDL-C) is recommended as a secondary target of therapy in patients with TG >200 mg/dl and takes into account the VLDL-C fraction [12]. However, non-HDL-C is superior to LDL-C in predicting CAD events regardless of whether TG is greater than or less than 200 mg/dl [6,11]. In order to more accurately assess the relationship between obesity and the lipid panel, we sought to describe the relationship between VFA, SFA, LDL-C, VLDL-C and non-HDL-C. Since increasing levels of VLDL-C are associated with increasing hepatic cholesterol synthesis [13,14], we also related VFA and SFA to cholesterol synthesis markers (desmosterol, lathosterol) and cholesterol absorption markers (cholestanol and sitosterol) to determine the relationship between cholesterol metabolism and visceral adiposity.

## Methods

### Study Population

We recruited patients with established vascular disease into this study. All patients had documented coronary artery disease (CAD), ischemic stroke or CAD risk-equivalents and thus represented a high-risk cohort eligible for aggressive lipid-lowering therapy. Eligible patients had to have at least one of the following: 1. CAD (positive angiogram or history of myocardial infarction) 2. peripheral vascular disease (ABI <0.9 or history of lower limb revascularization for atherosclerosis) 3. abdominal aortic aneurysm 4. carotid atherosclerosis with >50% narrowing 5. type II diabetes with age >50 and 3 additional risk factors (male sex, albuminuria, hypertension, HDL-C <40 mg/dl, TG >150 mg/dl, LDL-C >100 mg/dl, current smoking, diabetes duration >20 years) or 6. ischemic stroke. Study participants were in a clinically stable condition and were recruited from the vascular surgery outpatient department at the Royal Brisbane & Women's Hospital (RBWH). To ensure accurate assessment of endogenous cholesterol metabolism, patients treated with any statin, ezetimibe or stanol/sterol spreads at baseline were excluded. This study is approved by the RBWH research ethics committee (2005/006A) and all study participants gave informed consent.

### Patient Data

Patient demographic information was collected including age, sex, qualifying criterion, self-reported race, height, weight, waist and hip circumferences. Fasting blood samples were analyzed for baseline lipids, glucose, insulin, high sensitivity C-reactive protein (CRP), cholestanol, sitosterol, desmosterol and lathosterol. To determine insulin sensitivity, we used the Quantitative insulin sensitivity check index (QUICK Index) since this a superior linear correlate (r~0.8-0.9) of the reference standard glucose clamp than the Homeostasis model assessment (HOMA) model [15,16].

### MRI Measurement of Abdominal Fat Areas

MR imaging was performed with a Siemens Trio 3 T MRI system (Erlangen, Germany) using standard array coils with the subject supine. Breath-hold FISP images were centered on the L4-L5 intervertebral disc using standard localizer images with the following parameters: TR = 4 ms, TE = 2 ms, number of slices = 12, slice thickness = 8 mm, image matrix 256 × 256, field-of-view = 500 × 500 mm. The 4 slices that were best aligned with the L4-L5 disc (19, 20), were analyzed by a single operator (MRH) using the polygon region of interest in Escape Medical Viewer v3.2 to define visceral fat area (VFA) and subcutaneous fat area (SFA) as described previously [17]. Briefly, VFA and SFA were measured by fitting a spline curve to points on the border of the subcutaneous and visceral regions. Nonfat regions within the visceral region were also outlined with a spline fit and subtracted from the total visceral region.

### Laboratory Methodology

Fasting lipid profile was determined using standard hospital methods and LDL-C was indirectly measured using the Friedewald method. CRP was determined by high-sensitivity immunonephelometry. Insulins were measured by Chemilumnescent Immunoassay on a Beckman Coulter DxI800 as per the manufacturer's instructions. Cholestanol, sitosterol, desmosterol and lathosterol were measured using gas chromatography/ mass spectrometry (GCMS) (Agilent, Model 6890/ 5973 using Chemstation software). Internal standards were 5-α-cholestane for cholestanol, D7 sitosterol for sitosterol and D4 lathosterol for both lathosterol and desmosterol. Plasma (50 μL) was assayed with the addition of internal standards. The sample was then saponified at 60 degrees Celsius for 60 minutes using potassium hydroxide and extracted three times with hexane, each time centrifuging and combining organic layers. Combined organic layers were dried under nitrogen and the residue derivatised with bistrimethylsilyl trifluoroacetic anhydride/ 1% trimethylchlorosilane (BSTFA/TMCS) to form trimethylsilyl derivatives. The final derivatised extract (1 μL) was injected into the GCMS system and sterols separated using a capillary column DB 5 MS type (length 30 m, 0.25 mm internal diameter and 0.25 micron film thickness). Diagnostic ions specific for cholestanol, sitosterol, desmosterol, lathosterol and internal standards were monitored and the ratio of the sterol target ions to their respective internal standard ions was calculated and related to a calibration curve. The calibration curve was based on spiked normal plasma corrected for endogenous sterol levels using the technique of standard addition. Inter and intra-run coefficients of variation were 5% and 3% respectively. Linearity (r^2 ^= 0.998) was established beyond physiological values.

### Statistical Methods

The QUICK Index of insulin sensitivity for each subject was calculated as 1/[log (fasting insulin, μU/ml) + log (fasting glucose, mg/dl)] [15]. We first undertook univariate correlation with VFA or SFA as the dependent variable and the baseline fasting lipid panel, fasting glucose, fasting insulin and QUICK Index as independents. Skewed distributions were log-transformed. This was followed by correlations between VFA, SFA and various markers of cholesterol metabolism. All analyses were done with statistics software (SPSS 16).

## Results

Forty three patients were enrolled in this study. One patient was removed from this analysis because of hypertriglyceridemia (TG > 400 mg/dl), rendering indirect methods of LDL-C determination inaccurate leaving 42 patients in this analysis. Baseline characteristics, including baseline lipid panel, CRP, cholesterol synthesis markers (lathosterol, desmosterol) and cholesterol absorption markers (cholestanol, sitosterol) were available in all patients. The baseline characteristics of the 42 patients who are included in this analysis are shown in Table 1. Summary statistics for the baseline lipid panel and cholesterol metabolism markers are shown in Table 2. In order to explore the relationship between visceral fat area (VFA), subcutaneous fat area (SFA) and metabolic parameters, we undertook univariate correlation between VFA, SFA and components of the lipid panel, CRP, fasting glucose, insulin and the QUICK Index of insulin sensitivity. The results of univariate correlation are shown in Table 3. Concordant with previous reports, VFA is a more consistent correlate of metabolic parameters than SFA. SFA was not significantly correlated to VLDL-C, triglycerides or fasting glucose. Hence, in our cohort, VFA overall appears a superior correlate to the adverse metabolic consequences of obesity. A noteworthy finding was the significant negative correlation between LDL-C and VFA (r = -0.348, p = 0.024). Hence, while higher VFA has been independently associated with an increased risk of CAD, VFA is inversely correlated to LDL-C and thus patients with higher amounts of VFA may have lower LDL-C. Hence, this might explain the 'disconnect' between LDL-C and subsequent clinical events observed in patients with diabetes and hypertriglyceridemia [6,8,11].

**Table 1:** Patient Characteristics of Patients Included in the Analysis

	Mean ± SD or n (%)
Age	70 ± 8

Males	34 (81)

Females	8 (19)

Race	

White	41 (98)

Non-White	1 (2)

Height (cm)	172 ± 10

Weight (kg)	78 ± 19

Body Mass Index	26 ± 5

Waist (cm)	101 ± 16

Hip (cm)	101 ± 13

Diabetic	10 (24)

Metabolic Syndrome by ATP III Criteria	22 (52)

Fasting Glucose (mg/dl)	115 ± 37

Fasting Insulin (mU/L)	7 ± 4

QUICK	.25 ± .02

Visceral Fat Area (cm^2^)	202 ± 112

Subcutaneous Fat Area (cm^2^)	239 ± 115

Percent Visceral Fat Area	45 ± 12

Qualifying Criterion*	

Coronary Artery Disease	10 (24)

Peripheral Vascular Disease	23 (55)

Carotid Atherosclerosis >50%	12 (29)

Abdominal Aortic Aneurysm	10 (24)

Ischemic Stroke	8 (19)

Diabetes	10 (24)

*Patients frequently had >1 inclusion criterion

**Table 2:** Summary Population Lipids and CRP at Baseline

	Baseline value (mean ± SD)
Total Cholesterol (mg/dl)	212 ± 34

LDL cholesterol (mg/dl)	138 ± 33

HDL cholesterol (mg/dl)	45 ± 16

Non-HDL cholesterol (mg/dl)	167 ± 33

VLDL cholesterol	29 ± 14

Triglycerides (mg/dl)	146 ± 72

CRP (mg/L)	3.53 ± 2.47

Cholestanol (μmol/L)	7.03 ± 2.73

Desmosterol (μmol/L)	2.77 ± 0.94

Lathosterol (μmol/L)	5.88 ± 3.18

Sitosterol (μmol/L)	6.31 ± 3.65

Cholestanol:cholesterol ratio (μmol cholestanol/mmol cholesterol × 100)*	126 ± 40

Desmosterol:cholesterol ratio (μmol desmosterol/mmol cholesterol × 100)	51 ± 16

Lathosterol:cholesterol ratio (μmol lathosterol/mmol cholesterol × 100)	109 ± 62

Sitosterol:cholesterol ratio (μmol sitosterol/mmol cholesterol × 100)	112 ± 57

*this format is adopted for ease of comparison with previous reports; synthesis and absorption markers are standardized to total cholesterol.

**Table 3:** Univariate Correlations of Visceral Fat Area, Subcutaneous Fat Area with Components of the Lipid Panel, Glucose, Insulin and the QUICK Index

	Visceral Fat Area; Pearson's r, (p-value)	Subcutaneous Fat Area; Pearson's r, (p-value)
Total Cholesterol	-.306 (.049)	-.142 (.370)

LDL Cholesterol	-.348 (.024)	-.068 (.668)

HDL Cholesterol	-.361 (.019)	-.339 (.028)

Non-HDL Cholesterol	-.139 (.381)	.029 (.857)

VLDL Cholesterol	.503 (.001)	.217 (.168)

Triglycerides	.499 (.001)	.226 (.151)

CRP	.111 (.484)	.000 (.995)

Fasting Glucose	.522 (<.001)	.111 (.486)

Insulin	.664 (<.001)	.682 (<.001)

QUICK Index	-.723 (<.001)	-.592 (<.001)

Moreover, VFA was a strong positive correlate of VLDL-C which is predictive of coronary events independently of LDL-C [6,11]. VLVL-C is not routinely considered for risk assessment in the current ATPIII guidelines. Since increasing VLDL-C is indicative of increased hepatic synthesis of cholesterol, we tested this more formally by assessing the relationships between VFA, SFA and cholesterol synthesis markers (desmosterol, lathosterol) and cholesterol absorption markers (cholestanol and sitosterol). The results of this analysis are shown in Table 4. While neither VFA nor SFA correlated with sitosterol (an absorption marker), VFA correlated negatively with cholestanol (an absorption marker) and positively with synthesis makers desmosterol and lathosterol or their ratios. Hence, VFA is consistently associated with another metabolic trait of obesity, increased cholesterol synthesis and decreased cholesterol absorption [18], while SFA is not. This shift in phenotype is reflected by the positive correlation between VFA and VLDL-C and this shift in cholesterol metabolism is not reflected when LDL-C is considered in isolation to guide lipid-lowering therapy.

**Table 4:** Univariate Correlations of Visceral Fat Area, Subcutaneous Fat Area and Markers of Cholesterol Synthesis (Desmosterol, Lathosterol) and Cholesterol Absorption (Cholestanol, Sitosterol)

	Visceral Fat Area; Pearson's r, (p-value)	Subcutaneous Fat Area; Pearson's r, (p-value)
Cholestanol	-.433 (.004)	-.270 (.084)

Cholestanol:cholesterol ratio; CCR	-.357 (.020)	-.236 (.132)

Desmosterol	.264 (.092)	.158 (.317)

Desmosterol:cholesterol ratio; DCR	.421 (.006)	.232 (.140)

Lathosterol	.404 (.008)	.528 (<.001)

Lathosterol:cholesterol ratio; LCR	.454 (.003)	.548 (<.001)

Sitosterol	-.248 (.114)	-.273 (.080)

## Discussion

Our most important finding is the negative correlation between VFA, which independently predicts the development of CAD [1], and LDL-C which is the primary therapeutic target recommended by the ATPIII guidelines for the prevention and treatment of coronary disease. This observation might explain the 'disconnect' between LDL-C and subsequent clinical events which has been observed in patients with diabetes and hypertriglyceridemia [6,8,11]. However, VFA was a strong positive correlate of VLDL-C which is predictive of coronary events independently of LDL-C [11]. Non-HDL-C, which for practical purposes is the sum of LDL-C and VLDL-C, did not correlate with VFA or SFA and thus may be more useful in patients with higher degrees of visceral adiposity since it is not spuriously decreased as LDL-C is. VFA correlated negatively with cholestanol, a cholesterol absorption marker and positively with synthesis makers, desmosterol and lathosterol or their ratios. This shift in phenotype with obesity concordant with the positive association between VFA and VLDL-C; an effect that is not captured by isolated consideration of LDL-C. To provide an illustrative example of this, we compared the LDL-C in patients with and without the metabolic syndrome in our cohort. The metabolic syndrome is associated with an increased risk of cardiovascular events in vascular populations similar to ours and is therefore a clinically relevant label [19-22]. Patients with the metabolic syndrome in our cohort had greater visceral fat areas (263 vs 134 cm^2^, P < 0.01) but lower LDL-C (126 vs 153 mg/dl, p < 0.01) compared to patients without the metabolic syndrome. Hence patients in our cohort with the metabolic syndrome who are at an increased risk for future cardiovascular events may be incorrectly deemed lower risk by isolated consideration of LDL-C on the standard lipid panel.

Several larger studies have correlated VFA to total cholesterol and have shown both significant positive and negative correlation of total cholesterol and VFA [4,23]. However, total cholesterol is no longer the primary target of lipid-lowering therapy. Studies that have related LDL-C to obesity measures such as BMI have also shown both positive and negative associations. While excess body weight has been consistently associated with increases in TG, VLDL-C and decreased HDL-C, the effects of body weight on LDL-C have been variable [24-28]. In our study, the 'gold standard' measure of adiposity, the visceral fat area, in fact showed a negative correlation with LDL-C. The implications of this finding are that high-risk patients with large amounts of visceral adipose tissue may not qualify for drug therapy based on the current ATPIII guidelines, despite being at increased risk for coronary disease [1]. LDL-C has been criticized as an increasingly obsolete therapeutic target in the face of a growing obesity epidemic and non-HDL-C has been proposed as a more appropriate therapeutic target which incorporates the VLDL-C fraction and therefore better capitulates shifting cholesterol metabolism with obesity [6]. In our cohort, non-HDL-C did not correlate significantly with VFA and therefore may be a more appropriate target of therapy than LDL-C that can be applied 'across the board' in both lean and obese individuals. However, since non-HDL-C did not correlate positively with VFA, it is perhaps not the ideal therapeutic target since it did not capture the increased risk associated with increased visceral adipose tissue.

ApoB, which reflects the total number of atherogenic lipid particles, is widely regarded as the 'gold standard' for assessing cardiovascular risk and guiding therapy [29,30]. ApoB correlates positively with visceral fat area and may therefore best capture the increased cardiovascular risk with increasing visceral adiposity [23]. ApoB concentrations added to the predictive value of LDL-C in the Quebec Heart Study which was a prospective cohort followed for 13 years [31]. While non-HDL-C was a superior correlate of apoB than LDL-C, there remains some discordance between non-HDL-C and ApoB [32,33]. While non-HDL-C is superior to LDL-C in cardiovascular risk prediction, it may not fully reflect risk associated with the smaller LDL particles which is a hallmark of obesity and insulin resistance [34]. Unfortunately, ApoB assays are not routine in Australia or in many parts of the world and we were unable to assess ApoB in our cohort. On a World-wide basis, the standard lipid panel remains the mainstay of lipid assessment. In this context, non-HDL-C as a therapeutic target may be superior to LDL-C and has the advantage of not being spuriously low in patients with greater amounts of visceral adipose tissue and an increased risk for cardiovascular events.

## Conflicts of interests

The authors declare that they have no competing interests.

## Authors' contributions

MRH: designed the study, recruited the patients, primarily extracted the data from the MRI images, analyzed the data and wrote the manuscript. GC, RB and CM contributed to imaging protocols and imaging acquisition AC contributed to imaging protocols and supervised patient imaging. All authors read and approved the final manuscript.
